# Some hematological and biochemical parameters of different goat breeds in Sultanate of Oman “A preliminary study”

**DOI:** 10.14202/vetworld.2017.461-466

**Published:** 2017-04-30

**Authors:** Shahab Al-Bulushi, Turke Shawaf, Afaf Al-Hasani

**Affiliations:** 1 Department of Clinical Studies, College of Veterinary Medicine, King Faisal University, PO Box 400 Al-Hasa, 31982, Saudi Arabia; 2 Department of Animal and Veterinary Sciences, College of Agricultural and Marine Sciences, Sultan Qaboos University, PO Box 34, Al-Khod 123, Sultanate of Oman

**Keywords:** biochemical, breeds, goat, hematological, parameters, Omani

## Abstract

**Aim:**

In Sultanate of Oman, goats are considered as one of the most important livestock in which there are many breeds of goat such as Batinah, Jabali, Dhofari, Jabal Al-Akhdar, Sahrawi, and Sahrawi Musandam. Little hematological and biochemical information is known on Omani goat breeds; therefore, the main purpose of this study was to determine reference baseline data regarding hematological and biochemical values of different Omani goat breeds.

**Materials and Methods:**

A total of 30 healthy animals of different Omani goat breeds (Jabali, Jabal Al-Akhdar, Sahrawi, and Sahrawi Musandam) were selected randomly from different areas in Sultanate of Oman. The blood samples were collected from the jugular vein into two tubes for blood hematology and biochemical analysis. Statistical analysis was applied by using GraphPad Prism 7 software to calculate the minimum and maximum values to determine the range, mean, standard deviation of the mean and the p value.

**Results:**

No statistically significant variation in most hematological and biochemical parameters was found among the Omani goat breeds. The results of blood hematology revealed that the mean white blood cells (14.6±3.32 ×10^3^/µL), and the percentage of neutrophils in Omani goats (60.87±8.46%) were higher than that in most goat breeds. Higher values of red blood cells (12.8±1.28 ×10^6^/µL), hemoglobin (10.4±1.92 g/dl), hematocrit (38.29±4.06%), and lower values of mean corpuscular HGB concentration (27.05±3.5 g/dl) were observed in Omani goat breeds comparing to that in the other goat breeds. Lower values of total bilirubin (0.22±0.03 mg/dl), blood urea nitrogen (14.62±2.66 mg/dl), and cholesterol (48.58±19.05 mg/dl) were found in Omani goat breeds when compared to that of the other goat breeds.

**Conclusion:**

The obtained results are considered as the first values to be published for the different Omani goat breeds. This study is considered as preliminary study which can be used as a reference for further studies to determine reference values for the studied breeds to aid the veterinarians in the interpretation of the laboratory data and for the selection of the appropriate treatment.

## Introduction

Goats are considered as ideal animals to keep due to their high ability to survive under severe conditions and due to their ability to produce high-quality meat and milk [1]. In Sultanate of Oman, goats are considered as one of the most important livestock. In 1993, out of 1.35 million livestock animals, there were about 850 thousand goats [2]. Most of the goats in Oman are reared by using traditional methods in which they graze and browse in the available lands of the country [3]. In Oman, there are many breeds of goat such as Batinah, Jabali (also known as Rahbi), Dhofari, Jabal Al-Akhdar, Sahrawi (also known as Badawi goats), and Sahrawi Musandam (also known as Hawi goats). Three of these breeds are more common and numerous which are Jabal Al-Akhdar, Batinah, and Dhofari [3]. Due to the limited information about the local Omani goat breeds about their blood hematology and blood chemistry, it is essential to conduct such a research especially because local people prefer them more than the commercial breeds.

Hematological, biochemical, and mineral profiles are important to be determined because they provide valuable information about the breed, sex and animals health status [4]. There is considerable information about the normal parameters of blood of the domestic animal species, but the values are expected to vary according to the breeds, different environmental factors and the different methods of management [5]. The physiological adaptation and the systemic relationship are widely determined using the hematological values [6].

The biochemical profile shows some changes and the blood plasma components which varies according to the growth requirements, breed, ages [7], environmental factors, management conditions [8], sexual maturity [9], and the productivity of the animals [4].

This research was conducted to compare between the blood hematology and blood chemistry of different Omani goats breed as a preliminary study, comparing with some other breeds such as West African dwarf (WAD), Barbari, Black Aardi, and Sokoto red goats and to compare the obtained results with the standard values.

## Materials and Methods

### Ethical approval

The experiments on animals were approved by Livestock Research Center, Ministry of Agriculture and Fisheries, Sultanate of Oman.

### Animal sampling

A total of 30 animals of different Omani goat breeds (Jabali, Jabal Al-Akhdar, Sahrawi, and Sahrawi Musandam) were collected randomly from different areas in Sultanate of Oman. All of the animals were considered clinically healthy animals at the time of sampling and all of them were fed on the same diet.

### Blood sampling

Blood samples were collected from the jugular vein into two tubes (Guangzhou Improve Medical, China), in which one contained ethylenediaminetetraacetic acid for blood hematology and the other tube contains no anticoagulants (Becton Dickinson, Franklin Lakes, USA) for the biochemical analysis. All of the samples were transferred into the laboratory as quick as possible in ice.

### Blood hematology

Blood hematology prepared by using a special blood lysing buffer approved for goat hematology (Concentrated Lysing Reagent, SEAC, Florence, Italy). All the samples were analyzed within 45 min after collection by using CELL-DYN 3700 analyzer for the total red blood cells count (RBC), total and differential white blood cells count (WBC), hemoglobin (HGB), hematocrit (HCT), mean corpuscular volume (MCV), mean corpuscular HGB (MCH), MCH concentration (MCHC), red cell distribution width, and mean platelet volume.

### Serum biochemistry

The samples were left to clot and then centrifuged at 3000 rpm for 15 min, and the serum is collected. Serum was kept frozen at −20°C until it was used for the biochemical analysis. Serum biochemistry was carried out by using Vet Scan VS2 analyzer (ABAXIS, USA) for the mammalian liver profile which includes the following parameters: Albumin (ALB), alanine aminotransferase (ALT), alkaline phosphatase (ALP), bile acids (BA), cholesterol (CHOL), gamma glutamyl transferase (GGT), total bilirubin (TBIL), and blood urea nitrogen (BUN).

### Statistical analysis

For the statistical analysis, GraphPad Prism 7 software is used to calculate the minimum and maximum values to determine the range, mean, standard deviation of the mean also Shapiro–Wilk normality test was used to evaluate the normal distribution of the values and the p value significant were determined (p<0.05).

## Results and Discussion

Table-1 showed the values (means±standard deviation [SD] or standard error [SE]) of the blood hematology of the Omani goats (Jabali, Jabal Al-Akhdar, Sahrawi, and Sahrawi Musandam) in compare with some other breeds that are found in different regions worldwide (Barbari, Black Aardi, Damascus [7], Kano brown [8] with the determination of the significance (p<0.05) and it also contains the reference values that are determined by Feldman [10]).

**Table-1:** Hematological values (mean±SD or SE) in Omani goat breeds and different breeds of goats.

Parameter	Breed (Mean±SD)					p values	Reference value***

	Omani breeds N=30	Barbari*	Black Aardi*	Damascus*	Kano brown**		

					Mean±SE		
WBC (×10^3^/µL)	14.6±3.32	12.88±4.83	12.20±2.97	8.05±2.06		0.53	3-13
NEU %	60.87±8.46	56.80±5.23	42.90±5.80	39.90±38.18	26±1.46	0.73	30-48
LYM %	32.32±7.29	36.35±4.45	49.70±1.70	47.15±27.36	72.5±1.65	0.46	50-70
MONO %	3.34±1.88	4.52±2.11	3.73±2.56	5.61±4.99	0	0.43	0-4
EOS %	2.78±1.89	2.06±1.51	2.56±1.14	6.04±4.33	1.5±0.02	0.09	1-8
BASO %	0.67±0.37	0.27±0.15	1.08±0.40	1.36±1.52	0	0.84	0-1
RBC (×10^6^/µL)	12.8±1.28	12.15±1.06	11.20±0.42	10.44±0.93	NA	0.89	8-18
HGB (g/dL)	10.4±1.92	9.97±2.73	8.43±0.38	6.97±0.26	9.75±0.49	0.38	8-12
HCT %	38.29±4.06	16.80±2.26	14.55±0.64	12.70±1.55	43.4±0.92	0.098	22-38
MCV (fL)	30.08±2.42	13.80±0.56	12.95±0.07	12.15±0.49	100.9±1.73	0.006	16-25
MCH (pg)	8.09±0.83	8.14±1.53	7.53±0.63	6.70±0.33	23.75±0.77	0.001	2.20-8
MCHC (g/dL)	27.05±3.5	58.90±8.63	58.05±5.16	55.25±4.88	23.8±0.37	0.041	30-36
RDW %	35.86±3.57	34.05±3.04	35.25±6.29	31.35±3.32	NA	0.47	NA

* Mohammed *et al*. [23],

** Njidda *et al*. [16],

*** Feldman *et al.* [10].

NA=Not available, WBC=White blood cells, NEU=Neutrophils, LYM=Lymphocytes, MONO=Monocytes, EOS=Eosinophils, BASO=Basophils, RBC=Red blood cells, HGB=Hemoglobin, HCT=Hematocrit, MCV=Mean corpuscular volume, MCH=Mean corpuscular hemoglobin, MCHC=Mean corpuscular hemoglobin concentration, RDW=Red cell distribution width, SD=Standard deviation, SE=Standard error

The results of blood hematology of the breeds that were used in the study revealed that there was no significant variation in most of the hematological parameters except for three parameters which are MCV (p=0.006), MCH (p=0.001), and MCHC (p=0.041).

According to the obtained results, the Omani goats had higher WBCs and neutrophils percentage than that reported in Babari, Black Aardi, Damascus, Kano brown and Nigerian Sahel goats [7,8,11]. However, the WBCs in the Omani goats were also higher than the reference range that is reported by Feldman *et al*. [10].

In contrast, lymphocytes percentage was lower in the Omani than that reported in the other breeds [7,8], which could be due to the extremely high percentage of neutrophils in Omani goat breeds.

The percentage of monocytes, eosinophils and basophils agreed with the data reported on the Barbari and black Aardi breeds [7], but slightly lower than the reported value in Damascus breed [7]. However, these values were within the normal range reported by Feldman *et al*. [10].

The values of RBC, HGB, and HCT in our study were within the normal range that is reported by Feldman *et al*. [10]. Similar results also observed in Barbari Black Aardi breeds [7]. In contrast, the values of RBCs, HGB, and HCT were higher than that reported in the Damascus breed [7]. Higher values of MCV and MCH and lower values of MCHC were observed in Omani goat breeds comparing to that in the other goat breeds [7-11].

The results of some blood chemistry parameters (means±SD or SE) of the Omani goat breeds in this study are listed in Table-2 which includes the fallowing parameters: ALB, ALT, ALP, BA, CHOL, GGT, TBIL, and BUN. These results were compared with that of other goat breeds [11,12] with significance determination (p<0.05) and the reference range in goats [13].

**Table-2:** Serum biochemistry values of different goat breeds were compared together.

Parameter	Breed (Mean±SD)			Mean±SE		p values	Reference value****
	
	Omani N=30	Saanen*	Sahel*	Sokoto red**	WAD***		
ALP (IU/L)	80.91±27.34	103.22±15.10	243.41±8.5	209.38±17.56	63.25±6.9	0.25	93-387
ALT (IU/L)	22.83±4.34	49.28±3.32	NA	NA	5.3±0.7	0.78	6-19
GGT (IU/L)	51.85±9.13	53.27±4.0	NA	NA	NA		20-56
BA (µmol/L)	54.93±34.34	NA	NA	NA	NA		NA
TBIL (mg/dl)	0.22±0.03	0.1	2.34±0.12	2.26±0.25	2.07±0.17	0.038	0-0.1
ALB (g/dl)	3.07±0.25	2.76±0.48	4.25±0.11	4.43±0.19	2.8±0.1	0.09	2.7-3.9
BUN (mg/dl)	14.62±2.66	33.16±13.45	48.45±1.51	47.81±3.11	38.0±1.7	0.34	10-20
CHOL (mg/dl)	48.58±19.05	71.18±2.04	111.76±4.32	106.48±8.7	47.47±4.5	0.19	80-130

* Elitok [14],

** Njidda *et al*. [16],

*** Opara *et al*. [15],

**** Latimer [13].

NA=Not available, ALP=Alkaline phosphatase, ALT=Alanine aminotransferase, GGT=Gamma glutamyl transferase, BA=Bile acids, TBIL=Total bilirubin, ALB=Albumin, BUN=Blood urea nitrogen, CHOL=Cholesterol, WAD=West African dwarf, SD=Standard deviation, SE=Standard error

Due to the difference in the units of biochemical parameters in the studies on the Babari, Black Aardi, Damascus and Kano brown goats that have been used to compare within the hematological parameters, we used some other breeds from different studies to compare the blood chemistry of Omani goat breeds with. However, there were no significant variations in the biochemical values except for TBIL (p=0.038).

The results of our study showed lower ALP values than that reported in Sokoto red, Sahel and Saanen goats [11,12,14]. However, the values of ALP were slightly higher than that reported in WAD goats [15]. ALP constitutes a large group of isoenzymes, which plays important roles in the transportation of sugar, phosphate and it originates from different tissues such as liver, bone, placenta, and intestine [11].

Serum GGT in this study was slightly lower than that reported by Elitok, 2012 [16] on Saanen goat breed but it was within the reference range [13].

In our study, mean ALB concentration was lower than that reported in Sahel and red sokoto breeds but it was slightly higher than that reported in Saanen and WAD goat breeds. The differences in ALB values may be caused by liver diseases, malnutrition and dehydration [16].

Lower values of TBIL, BUN and CHOL were found in Omani goat breeds when compared to that of the other goat breeds [17,15,12,14]. Lower concentration of BUN is an indication of the dietary protein level or liver chronic diseases [18].

Table-3 showed the range and the mean±SD values of the blood hematology of the Omani goat breeds with the determination of the significance values (p<0.05), and the results were compared with the reference value mentioned by Feldman *et al*. [10].

**Table-3:** Hematological values of different breeds of goats of Oman with the reference value of goats by Feldman.

Parameter	Breed								p values	Reference value*

	Jabali N=7		Sahrawi N=10		Sahrawi Musandam N=9		Jabel Al Akhdar N=4	
			
	Mean±SD	Range	Mean±SD	Range	Mean±SD	Range	Mean±SD	Range
WBC (K/µL)	14.57±2.23	11.5-17.6	15.44±4.92	11.7-28.1	16.84±2.63	12.9-21.2	11.58±3.50	9.26-16.8	0.73	3-13
NEU %	61.8±5.05	56.6-71.4	62.71±10.22	49.9-80.4	52.19±12.53	36.8-74.6	66.78±6.03	60.5-74.5	0.45	30-48
LYM %	32.65±5.37	21.9-36	29.7±8.87	15.7-40.9	38.71±11.82	24.4-54.9	28.25±3.12	24-31.5	0.51	50-70
MONO %	2.86±0.88	1.26-3.71	3.174±1.95	0.64-5.92	3.62±2.11	0.37-6.24	3.72±2.57	0.92-6.19	0.52	0-4
EOS %	2.11±1.58	0.75-5.18	3.67±2.91	1.56-11.7	4.94±2.96	0.43-8.92	0.40±0.13	0.23-0.55	0.94	1-8
BASO %	0.60±0.18	0.31-0.88	0.73±0.38	0.17-1.46	0.53±0.28	0.24-1	0.81±0.65	0.31-1.78	0.79	0-1
RBC (M/µL)	13.32±0.44	12.9-14	12.5±0.93	11.5-14.4	12.69±0.78	11-13.8	12.69±2.99	8.47-15.4	0.15	8-18
HGB (g/dL)	11±1.92	9.09-13.8	9.73±1.64	8.05-12.9	10.37±1.45	7.37-12.2	10.51±2.72	6.63-13	0.88	8-12
HCT %	39.77±2.91	37.4-43.7	36.76±3.34	32.5-43.7	37.4±3.42	33.5-43.2	39.25±6.58	32.3-47.6	0.45	22-38
MCV (fL)	29.78±1.29	28.4-31.5	29.49±2.32	26.5-33.4	29.5±1.53	27.1-31.4	31.55±4.52	28-38.1	0.02	16-25
MCH (pg)	8.22±1.21	6.82-10.1	7.78±1.10	6.77-10.6	8.15±0.72	6.71-9.1	8.22±0.31	7.83-8.48	0.03	2.20-8
MCHC (g/dL)	27.52±2.89	24-31.9	26.52±4.14	22.4-36.8	27.66±2.81	22-30.9	26.53±4.15	20.6-30.3	0.08	30-36
RDW %	35.15±3.57	30-40.7	33.19±1.46	31.2-35.3	34.94±2.53	31.6-39	40.18±6.71	35.7-50	0.27	NA

* Feldman *et al*. [10].

WBC=White blood cells, NEU=Neutrophils, LYM=Lymphocytes, MONO=Monocytes, EOS=Eosinophils, BASO=Basophils, RBC=Red blood cells, HGB=Hemoglobin, HCT=Hematocrit, MCV=Mean corpuscular volume, MCH=Mean corpuscular hemoglobin, MCHC=Mean corpuscular hemoglobin concentration, RDW=Red cell distribution width, SD=Standard deviation, SE=Standard error

The results of blood hematology of the Omani goat breeds revealed that there was no significant variation in most of the hematological parameters, while there was slight significant deference in both MCV (p=0.02) and MCH (p=0.03).

Higher mean values of WBCs and neutrophils percentage were observed in Jabali, Sahrawi and Sahrawi Musandam than that in the reference range that reported by Feldman *et al*. [10], while the mean WBCs value of Jabal Al-Akhdar goats was in agreement with Feldman *et al*. values [10]. The higher WBCs and neutrophils in this study may be indicative of activation of defense and immune to infections or toxic substances.

Lower percentage of lymphocytes was observed in all of the Omani breeds than the normal range reported by Feldman which could obviously contributed by the increase in the percentage of neutrophils.

The percentage of monocytes, basophils, and eosinophils which were comparable in all of the Omani goat breeds were in agreement with that of Feldman *et al*. [10]. However, lower eosinophil percentage in Jabal Al-Akhdar breed was observed in compare with the other Omani breeds.

Values of RBC, HGB, HCT, MCV, MCH and MCHC, which help to determine and classify anemia [19] were observed to be lower in the Sahrawi breed than the other Omani breeds. Low RBCs count also may be associated with iron deficiency, bleeding, anemia or some vitamin deficiency.

The value of HGB and HCT percentages were slightly higher in both Jabali and Jabal Al-Akhdar goats in comparing to that of Sahrawi and Sahrawi Musandam goats. However, the high values of HGB and HCT could be related to the high altitude of the originated place of these breeds and their need to oxygen.

The results of some blood chemistry parameters (mean±SD and range) with the determination of the significance (p<0.05) obtained from Omani goat breeds are shown in Table-4. All of the chemistry values were similar (p>0.05) among Omani breeds, except the values of ALT (p=0.03) and TBIL (p=0.04).

**Table-4:** Serum biochemistry values (mean±SD with p values) of different breeds of goats of Oman with the reference value of goats by Latimer 2011.

Parameter	Breed								p values	Reference value*

	Jabali N=7		Sahrawi N=10		Sahrawi Musandam N=9		Jabel Al Akhdar N=4			
			
	Mean±SD	Range	Mean±SD	Range	Mean±SD	Range	Mean±SD	Range		
ALP (IU/L)	95±33.12	53-125	71.67±26.67	35-104	68±14.99	45-88	89±34.6	62-128	0.38	93-387
ALT (IU/L)	23.71±8.07	17-39	23.67±5.04	19-33	24.29±3.09	20-28	19.67±1.15	19-21	0.03	6-19
GGT (IU/L)	40.86±8.07	26-51	53.67±11.34	41-69	53.57±10.01	40-70	59.33±7.09	53-67	0.35	20-56
BA (µmol/L)	57.43±45.5	5-115	41.5±21.99	16-75	40.14±31.89	9-105	80.67±38	53-124	0.3	N.A
TBIL (mg/dl)	0.22±0.05	0.2-0.3	0.2±0	0.2-0.2	0.22±0.05	0.2-0.3	0.23±0.06	0.2-0.3	0.04	0-0.1
ALB (g/dl)	2.9±0.37	2.4-3.4	3.21±0.22	2.8-3.4	3.15±0.14	3-3.4	3.03±0.29	2.7-3.2	0.77	2.7-3.9
BUN (mg/dl)	14.86±3.13	11-20	13.67±2.58	10-17	14.29±2.87	11-18	15.67±2.08	14-18	0.97	10-20
CHOL (mg/dl)	67.43±31.13	20-119	47±18.07	23-68	52.57±18.92	20-74	27.33±8.08	20-36	0.92	80-130

* Latimer, [13].

NA=Not available, ALP=Alkaline phosphatase, ALT=Alanine aminotransferase, GGT=Gamma glutamyl transferase, BA=Bile acids, TBIL=Total bilirubin, ALB=Albumin, BUN=Blood urea nitrogen, CHOL=Cholesterol, SD=Standard deviation, SE=Standard error

Serum ALP level was higher in Jabali and Jabel Al-Akhdar goats than that of Saharawi and Sahrawi Musandam. ALP level can influence by blood pH and diseases [20].

Higher values of BA, GGT and BUN and lower values of ALT and CHOL were observed in Jabal Al-Akhdar when compared to the other Omani breeds. It could be due to differences in breed, climate, animal housing, nutrition and subclinical diseases [21,22].

Interestingly the CHOL concentration was extremely lower in all of Omani breeds especially in Jabal Al-Akhdar breed (mean=27.33±8.08) in compare to the standard values, which indicates that this breed is better for human consumption.

## Conclusion

The obtained results are considered as the first values to be published for the different Omani goat breeds. This study is considered as preliminary study which can be used as a reference for further studies to determine reference values for the studied breeds to aid the veterinarians in the interpretation of the laboratory data and for the selection of the appropriate treatment.

## Authors’ Contributions

SA and TS conceived the study, performed the fieldwork, collected the samples, carried out the laboratory work, analyzed the data and drafted the manuscript, AA read and approved the final manuscript. All authors read and approved the final manuscript.
